# Revisiting the Clinical Importance of DPYD*9A (c.85T>C) Variant of Dihydropyrimidine Dehydrogenase (DPYD) Gene in Patients Treated with Fluoropyrimidine-Based Chemotherapy

**DOI:** 10.4172/2155-9929.1000e129

**Published:** 2018-06-18

**Authors:** Girijesh Kumar Patel, William Taylor, Seema Singh, Moh’d Khushman, Ajay P Singh

**Affiliations:** 1 Department of Oncologic Sciences, Mitchell Cancer Institute, University of South Alabama, Mobile, Alabama, USA; 2 Medical Oncology, Mitchell Cancer Institute, University of South Alabama, Mobile, Alabama, USA; 3 Department of Biochemistry and Molecular Biology, College of Medicine, University of South Alabama, Mobile, Alabama, USA

## Editorial

Gastrointestinal toxicity due to chemotherapeutic drugs is a common problem in cancer patients. Chemotherapy-related diarrhea is most commonly described with fluoropyrimidines (fluorouracil and capecitabine) and irinotecan. Fluoropyrimidines are widely used for the treatment of gastrointestinal (GI) tract tumors and also in other solid malignancies such as breast and head and neck cancers [1,2] and are relatively well tolerated, however, around 5% to 10% of the treated patients develops severe, potentially life threatening toxicity such as GI toxicity, skin toxicity, myelosuppression and neurotoxicity [3]. Early identification of patients at risk of developing fluoropyrimidines-induced toxicity by upfront screening might allow dose reduction or selection of an alternative chemotherapy regimen. The two well-studies predictive markers for fluoropyrimidines-related toxicity are dihydropyrimidine dehydrogenase (DPD) and thymidylate synthase (TS) enzymatic activity.

The DPD is a rate-limiting enzyme out of three enzymes in fluoropyrimidine metabolic pathways. The partial or complete deficiency of DPD activity has been shown to increase severe or fatal toxicity. To date, over 128 mutations and polymorphisms in DPD encoding gene (DPYD) has been reported [4,5]. Among those, 3 variants (DPYD*2A, DPYD*13 and DPYD*9B) have been shown to be associated with reduced DPD activity and thus enhanced toxicity in patients treated with fluoropyrimidine-based chemotherapy. Some research group also reported the suppressed activity due the aberrant methylation of the DPYD promoter region acted as one of the repressors of DPYD expression at transcriptional level and affected sensitivity to 5-FU in cancer cells [6,7].

The correlation between DPYD*9A (c.85T>C) genotype and DPD deficiency clinical phenotype is controversial [8,9]. In some studies, in patients with GI malignancies, DPYD*9A (c.85T>C) variant was associated with fluoropyrimidines-associated toxicity. Patients experienced diarrhea (p<0.05) and hand foot syndrome (HFS) (p<0.05) [8,10,11]. Other clinical studies reported that there is no correlation between DPYD*9A (c.85T>C) genotype and DPD deficiency clinical phenotype. Based on the current limited knowledge, the 2017 updated Clinical Pharmacogenetics Implementation Consortium (CPIC) guideline for DPD genotype and fluoropyrimidine dosing, it was stated that the DPYD*9A (c.85T>C), among other variants, doesn’t affect DPD activity in a clinically relevant manner [12-14]. So, the reference laboratories either did not perform DPYD*9A genotyping or have stopped DPYD*9A genotyping and limited genotyping to high-risk variants (DPYD*2A, DPYD*13 and DPYD*9B) only.

Recently, our group has reported that DPYD*9A (c.85T>C) variant was the most common variant diagnosed in our cohort and a genotype-clinical phenotype correlation was noticeable. All patients who received full dose fluoropyrimidines experienced grade 3-4 diarrhea [15,16]. Other group have also reported the correlation between DPYD*9A (c.85T>C) and grade 3-4 toxicity [8,10,11]. Moreover, the Ti et al. [17] reported that the (DPYD*9A T/T and T/C genotypes accounted for 85.7% and 14.3% in colorectal cancer, respectively and correlated with the toxicity [17]. These finding indicate the importance of genotyping of this variant to avoid the fluoropyrimidine-related toxicity and need to be considered upfront along with other high risk variants.

Fluoropyrimifines-related toxicity not only results in treatment interruption but also sometimes discontinuation, adversely affecting the quality of life and an increase in health care cost. Up front screening of DPD deficiency through genotyping for high-risk DPYD variants (DPYD *2A, *13 and *9B) only is suboptimal to predict fluoropyrimidine-related toxicity. Genotyping for DPYD*9A in addition to the high-risk DPYD variants represents a more comprehensive approach. Diagnosing of DPD deficiency upfront provide the treating oncologist the opportunity to avoid fluoropyrimifines-related toxicity. In patients who are heterozygous, oncologists are advised to start their patients on a reduced dose with subsequent titration or choosing an alternative regimen. In patients who are homozygous, fluoropyrimidines-based regimen should be avoided.

## References

[1] Ershler WB (2006) Capecitabine monotherapy: Safe and effective treatment for metastatic breast cancer. Oncologist 11: 325–335. 16614228 10.1634/theoncologist.11-4-325

[2] Vermorken JB, Remenar E, Van Herpen C, Gorlia T, Mesia R, (2007) Cisplatin, fluorouracil, and docetaxel in unresectable head and neck cancer. New Engl J Med 357: 1695–1704. 17960012 10.1056/NEJMoa071028

[3] Hoff PM, Ansari R, Batist G, Cox J, Kocha W, (2001) Comparison of oral capecitabine versus intravenous fluorouracil plus leucovorin as firstline treatment in 605 patients with metastatic colorectal cancer: Results of a randomized phase III study. J Clin Oncol 19: 2282–2292. 11304782 10.1200/JCO.2001.19.8.2282

[4] Offer SM, Fossum CC, Wegner NJ, Stuflesser AJ, Butterfield GL, (2014) Comparative functional analysis of DPYD variants of potential clinical relevance to dihydropyrimidine dehydrogenase activity. Cancer Res 74: 2545–2554. 24648345 10.1158/0008-5472.CAN-13-2482 PMC4012613

[5] Krishnamurthi SS (2014) Enterotoxicity of chemotherapeutic agents. Up to date; Waltham, MA, USA.

[6] Noguchi T, Tanimoto K, Shimokuni T, Ukon K, Tsujimoto H, (2004) Aberrant methylation of DPYD promoter, DPYD expression, and cellular sensitivity to 5-fluorouracil in cancer cells. Clin Cancer Res 10: 7100–7107. 15501990 10.1158/1078-0432.CCR-04-0337

[7] Zhang X, Soong R, Wang K, Li L, Davie JR, (2007) Suppression of DPYD expression in RKO cells via DNA methylation in the regulatory region of the DPYD promoter: A potentially important epigenetic mechanism regulating DPYD expression. Biochem Cell Biol 85: 337–346. 17612628 10.1139/o07-009

[8] Joerger M, Huitema ADR, Boot H, Cats A, Doodeman VD, (2015) Germline TYMS genotype is highly predictive in patients with metastatic gastrointestinal malignancies receiving capecitabine-based chemotherapy. Cancer Chemother Pharmacol 75: 763–772. 25677447 10.1007/s00280-015-2698-7

[9] Kleibl Z, Fidlerova J, Kleiblova P, Kormunda S, Bilek M, (2009) Influence of dihydropyrimidine dehydrogenase gene (DPYD) coding sequence variants on the development of fluoropyrimidine-related toxicity in patients with highgrade toxicity and patients with excellent tolerance of fluoropyrimidine-based chemotherapy. Neoplasma 56: 303. 19473056 10.4149/neo_2009_04_303

[10] Vreken PV, Van Kuilenburg ABP, Meinsma R, Van Gennip AH (1997) Dihydropyrimidine dehydrogenase (DPD) deficiency: Identification and expression of missense mutations C29R, R886H and R235W. Hum Genet 101: 333–338. 9439663 10.1007/s004390050637

[11] Van Kuilenburg AB, Vreken P, Riva D, Botteon G, Abeling NG, (1999) Clinical and biochemical abnormalities in a patient with dihydropyrimidine dehydrogenase deficiency due to homozygosity for the C29R mutation. J Inherit Metab Dis 22: 191–192. 10234617 10.1023/a:1005470524203

[12] Amstutz U, Farese S, Aebi S, Largiadèr CR (2009) Dihydropyrimidine dehydrogenase gene variation and severe 5-fluorouracil toxicity: a haplotype assessment. Pharmacogenomics 10: 931–944. 19530960 10.2217/pgs.09.28

[13] Gross E, Busse B, Riemenschneider M, Neubauer S, Seek K, (2008) Strong association of a common dihydropyrimidine dehydrogenase gene polymorphism with fluoropyrimidine-related toxicity in cancer patients. PloS One 3: e4003. 19104657 10.1371/journal.pone.0004003 PMC2602733

[14] Seek K, Riemer S, Kates R, Ullrich T, Lutz V, (2005) Analysis of the DPYD gene implicated in 5-fluorouracil catabolism in a cohort of Caucasian individuals. Clin Cancer Res 11: 5886–5892. 16115930 10.1158/1078-0432.CCR-04-1784

[15] Khushman MD, Patel GK, Hosein PJ, Laurini J, Cameron D, (2018) Germline pharmacogenomics of DPYD* 9A (c. 85T> C) variant in patients with gastrointestinal malignancies treated with fluoropyrimidines. J Gastrointest Oncol 9: 1–9. 29998006 10.21037/jgo.2018.02.03 PMC6006041

[16] Khushman MD, Hosein PJ, Laurini J, Cameron D, Clarkson DR (2018) Germline pharmacogenomics of DPYD* 9A (c. 85T> C) variant in patients with gastrointestinal malignancies treated with fluoropyrimidines. J Clin Oncol 36: 598. 10.21037/jgo.2018.02.03 PMC6006041 29998006

[17] Li GY, Duan JF, Li WJ, Liu T (2016) DPYD* 2A/* 5A/* 9A and UGT1A1* 6/* 28 polymorphisms in Chinese colorectal cancer patients. J Cancer Res Ther 12: 782–786. 27461651 10.4103/0973-1482.148685

